# Effect of Qingxin Kaiqiao Fang on Hippocampus mRNA Expression of the Inflammation-Related Genes IL-1*β*, GFAP, and A*β* in an Alzheimer's Disease Rat Model

**DOI:** 10.1155/2018/9267653

**Published:** 2018-02-18

**Authors:** Dan-Dan Mao, Wen-Yu Yang, Yan Li, Jian-Wei Lin, Shi-Yu Gao, Yi-Ru Wang, Hai-Yan Hu

**Affiliations:** ^1^The Second Clinical College, Wenzhou Medical University, Wenzhou 325003, China; ^2^The Second Affiliated Hospital and Yuying Children's Hospital of Wenzhou Medical University, 109 Xue Yuan Xi Road, Lu Cheng District, Wenzhou 325000, China

## Abstract

**Objective:**

To investigate the effects of QKF on expression of amyloid-beta (A*β*), interleukin-1 beta (IL-1*β*), and glial fibrillary acidic protein (GFAP) using a rat model of AD.

**Materials and Methods:**

Fifty-six male Sprague-Dawley rats were randomly divided into seven groups (eight rats each): control group, sham-operated group, AD model group, groups of AD rats administered with low, medium, and high doses of QKF, and the donepezil group. AD was established by bilateral injection of *β*-amyloid (A*β*) 1–40 into the hippocampus. Two days after AD was established, drugs were administered by gavage. After 14 days of treatment, we used RT-PCR, Western blotting, and immunohistochemistry to measure the transcript expression and protein abundance of A*β*, IL-1*β*, and GFAP, and methenamine silver staining was used to detect amyloid protein particle deposition.

**Results:**

Compared to the control group, the rats from the AD model group showed significantly greater expression levels of A*β*, IL-1*β*, and GFAP. However, these differences in expression were abolished by treatment with QKF or donepezil.

**Conclusion:**

QKF possesses therapeutic potential against AD because it downregulated A*β*, IL-1*β*, and GFAP in the hippocampus of AD rats. Future studies should further examine the mechanisms through which QKF produces its effects and the consequences of long-term QKF administration.

## 1. Introduction

Alzheimer's disease (AD) is a common neurological degenerative disease among the elderly. It is characterized by progressive declines in memory and cognitive function and has an incidence rate of 10% to 30% in people over 85 years of age [1]. AD eventually leads to memory and cognitive disorders, daily behavior disorders, and dementia, and the incidence of AD increases with age. The main pathological features of AD are loss of neurons, senile plaque (SP), and neurofibrillary tangles (NFT) [2]. The core component of SP is beta-amyloid (A*β*), which has been shown to play an important role in the progression of AD [3, 4]. Glial cell proliferation and excessive cytokine production are involved in the formation of SP and NFT [5, 6], suggesting the involvement of the immune response. Because there are currently no approved effective AD treatments, compounds from traditional Chinese medicine offer a basis from which to discover new AD treatments.

Qingxin Kaiqiao Fang (QKF) is a component of the traditional Chinese medicine Man Jian, which is based on a recipe from the medical book Jingyue Quanshu written by Zhang Jing-yue during the Ming Dynasty. QKF has been used as a treatment for AD for many years and produces remarkable effects on early symptoms such as cognitive dysfunction and behavioral and psychological symptoms [7–9]. It is currently unknown whether brain inflammation in AD patients is the cause of the disease or a secondary phenomenon, but A*β* is known to promote astrocyte-mediated inflammatory responses and thus activate signaling pathways that could lead to neurodegeneration. This study investigated the potential of QKF as a treatment for AD by measuring the expression of A*β*, IL-1*β*, and GFAP in the hippocampus of rats in AD model.

## 2. Methods

### 2.1. Animals

Sprague-Dawley (SD) rats of specific pathogen free (SPF) grade (*N* = 56), weighing 250 ± 20 g, were purchased from the Beijing Vital River Laboratory Animal Technology Co., Ltd., China (certification number SCXK 2012-0001; Beijing). Rats were bred in Wenzhou Medical University Laboratory Animal Center (a qualified facility meeting clean experimental animal feeding standards). Rats were housed in standard laboratory cages with a 12 h light and dark cycle along with free access to food and water, and all animal experiments were performed in accordance with the ethical requirements approved by the Chinese Association of Accreditation of Laboratory Animal Care.

### 2.2. Drugs and Reagents

QKF is composed of* Radix Rehmanniae*,* Radix Ophiopogonis*,* Radix Paeoniae*,* Herba Dendrobii*,* Cortex Moutan Radicis*,* Poria Cocos*,* Pericarpium Citri Reticulatae*,* Rhizoma Anemarrhenae*,* Rhizoma Acori Tatarinowii*, and* Sophorae flavescentis*. These components were provided by the Second Affiliated Hospital of Wenzhou Medical University and verified by the Department of Chinese Materia Medica of Wenzhou Medical University. To make 1 g/mL drug stocks, the raw herbs were decocted with appropriate amounts of water, extracted twice, filtered, and concentrated. The drug stocks were kept at 4°C. Donepezil was used as a positive control and was purchased from the Eisai Pharmaceutical Co., Ltd. (Suzhou, China) (Number: 100223A).

A*β* 1–40 and DMSO were purchased from Sigma-Aldrich (St. Louis, MO, USA). The DAB reagent kit was obtained from Zymed (San Diego, CA, USA). Trizol reagent was bought from Shanghai ShengGong Biological Engineering Co., Ltd. Reverse transcriptase and SYBR green were obtained from Bioneer (Shanghai, China). All PCR primers were obtained from Dalian Treasure Biological Engineering Co. Ltd. Rabbit anti-mouse A*β*, GFAP, and IL-1*β* antibodies were obtained from Bioworld Technology, Inc. (St. Louis Park, MN, USA). Chloral hydrate was obtained from Sinopharm Chemical Reagent Co., Ltd. (Shanghai, China) and the BCA protein assay kit was obtained from Pierce (Rockford, IL, USA).

### 2.3. Animal Model and Groups

Aggregated A*β* 1–40 was prepared following the manufacturer's instructions. AD was established in rats by bilateral injection of A*β* 1–40 fragments into the hippocampus using stereotaxic methods [10]. In brief, eight of the 56 rats were randomly chosen for the control group (0 + NS). The other 48 rats were anesthetized via intraperitoneal injection of 10% chloral hydrate at a dose of 3-4 mL/kg. The rats were disinfected with 75% alcohol before the skulls were opened using a cranial drill. The bregma was located based on the rat brain atlas of Paxinos and Watson and used as a reference to drill a 1.5 mm opening on the right and left sides approximately 3 mm behind the bregma [11]. Rats were then subjected to microinfusion with 2*μ*L of ddH_2_O for the sham-operated group (*n* = eight, NS + NS) or 2*μ*L of 2.5*μ*g/*μ*L A*β* 1–40 (equivalent to 5*μ*g A*β*) using a 3-mm microsyringe. The day after the operation, the 40 A*β*-treated rats were randomly divided into five groups (eight rats in each group) and orally given drugs for 14 days. Rats in the normal control group (0 + NS), the sham-operated group (NS + NS), and the model group (A*β* + NS) were administered saline. Rats in the positive control group (A*β* + donepezil) were administered donepezil (1.67 mg/kg), and rats in the QKF groups were administered a low (4.75 mg/kg), medium (9.5 mg/kg), or high (19 mg/kg) dose of QKF (A*β* + L-FJ, A*β* + M-FJ, and A*β* + H-FJ, respectively). All QKF dosages were relevant to the treatment of human adults in clinical settings.

### 2.4. Hippocampus Collection

At the end of the experiment, the rats were anesthetized with 10% chloral hydrate. Brains were removed quickly and placed in 4% paraformaldehyde. The hippocampi were snap-frozen and stored at −80°C.

### 2.5. RT-PCR Analysis

Total RNA from the hippocampus was isolated with Trizol reagent and cDNA was synthesized with reverse transcriptase. Quantitative gene expression was measured by Real-time PCR. The data were analyzed using a LightCycler 480 II PCR cycler (Roche, Basil, Switzerland) based on Ct value and normalized to*β*-actin. All PCR primers were designed by the Shanghai Rui Jingsheng Biological Engineering Co., Ltd. (Table 1).

### 2.6. Western Blot Analysis

The frozen hippocampus tissue was ground and lysed in RIPA lysis buffer containing a protease inhibitor cocktail (Roche, Rockford, IL, USA). The protein was quantified by the BCA protein assay kit (Pierce, Rockford, IL, USA). Forty micrograms of total protein for each sample was separated by 10% SDS-PAGE and transferred to a polyvinylidene difluoride (PVDF) membrane. The PVDF membrane was blocked for 1 h at room temperature and incubated with primary antibodies overnight at 4°C. After several washes with TBST (Tris-buffered saline and Tween-20), the membranes were incubated with horseradish peroxidase-conjugated goat anti-rabbit secondary antibody at room temperature for 2 h. The protein bands were visualized using a chemiluminescence-based detection kit (Pierce, Rockford, IL, USA). The OD values of A*β*, IL-1*β*, GFAP, and*β*-actin were quantified using a Quantity One gel analysis system. The measurements were performed in triplicate for each rat.

### 2.7. Immunohistochemistry

The fixed brain tissues were removed, dehydrated with an alcohol gradient, impregnated with xylene transparent wax, embedded in paraffin, and sliced at a thickness of 5*μ*m, according to the manufacturer's instructions. An MIAS medical system (Media Cybernetics, Rockville, MD, USA) was used for image analysis. Each group was represented by six slices and each sample was observed at five horizons of the CA1 area of the hippocampus. All positive granules within view were identified, and density was calculated as the positive granules within the target area/total area of the statistical field.

### 2.8. Methenamine Silver Staining to Detect Amyloid Protein Particles

Methenamine silver primary liquid was mixed with an equal volume of sodium borate (1 : 1), boiled in a microwave oven, and kept at 60°C in a water bath. After sections were fully dewaxed, they were placed into 0.5% periodate and 8% chromic acid for 15 min and 30 min, respectively. After washing with distilled water, the sections were processed with 1% sodium metabisulfite solution for 1 min. Next, they were placed into preheated methenamine working solution and kept at 60°C in an incubator for 30–60 min, until the sections appeared as black particles on a brown background. Lastly, the sections were added to 1% gold chloride aqueous solution to tint for 2 min and then processed with 3% sodium thiosulfate solution for 3 min.

### 2.9. Statistical Analysis

All data were processed using SPSS 16.0 statistics software and expressed as mean ± standard deviation. First, a normality test was performed for all data (*P* < 0.1 signified normal distribution). Pair-wise comparisons were performed using the LSD test for homogeneous variance or Dunnett's test for nonhomogeneous variance. Differences were considered statistically significant when *P* < 0.05.

## 3. Results

### 3.1. General Health of Rats

In the control group, all rats had smooth fur, good food intake, quick reflexes, and normal weight gain. There were no deaths within 24 hours after surgery. The rats in the sham-operated group also demonstrated normal characteristics. The rats on which surgery was performed showed various degrees of malaise, low activity, loss of appetite, and weight loss, especially in the model group. After two weeks of medication, the treated rats recovered significantly, but there was no significant improvement in the model group.

### 3.2. Gene Expression of A*β*, IL-1*β*, and GFAP in the Hippocampus

**Figure 1:**
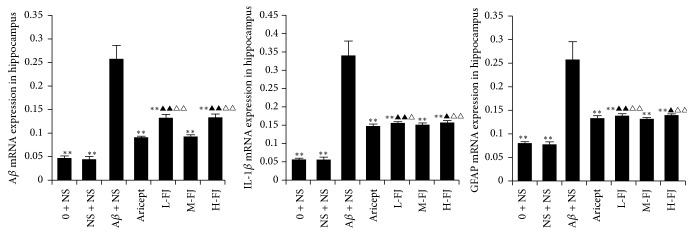
Gene expression of A*β*, IL-1*β*, and GFAP in the hippocampus. Compared to A*β* + NS group, ^*∗∗*^*P* < 0.01; compared to Aricept group, ^△△^*P* < 0.01, ^△^*P* < 0.05; compared to “M-FJ” group, ^▲^*P* < 0.05, ^▲▲^*P* < 0.01.

RT-PCR showed that there was no significant difference in the expression of A*β*, IL-1*β*, or GFAP between the control group and the sham-operated group. Expression of A*β*, IL-1*β*, and GFAP in the model control group was significantly increased compared to corresponding levels in the other groups (*P* < 0.05, *P* < 0.01). After treatment with donepezil or QKF, all observed changes in the expression of A*β*, IL-1*β*, and GFAP were reduced to different degrees (Figure 1).

### 3.3. Protein Levels of A*β*, IL-1*β*, and GFAP in the Hippocampus

**Figure 2:**
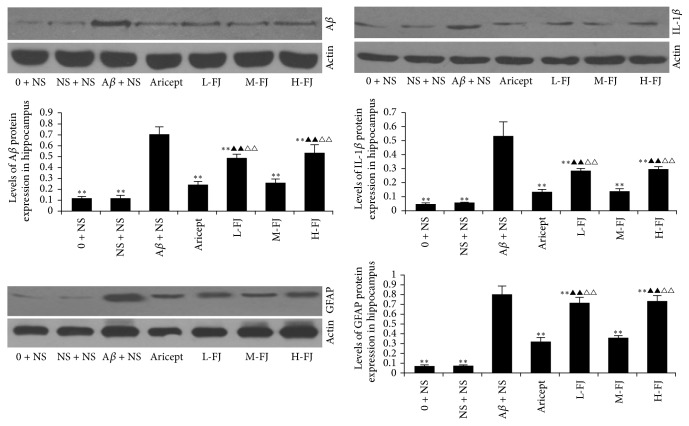
Protein expression of A*β*, IL-1*β*, and GFAP in the hippocampus. Compared to A*β* + NS group, ^*∗∗*^*P* < 0.01; compared to Aricept group, ^△△^*P* < 0.01; Compared to “M-FJ” group, ^▲▲^*P* < 0.01.

A*β*, IL-1*β*, and GFAP protein levels were significantly increased in the hippocampus of the AD model group, in comparison to the other groups (*P* < 0.01; Figure 2). After treatment with donepezil or QKF, changes in A*β*, IL-1*β*, and GFAP levels were reduced in comparison to the other groups. Of the QKF treatments, a dose of 9.5 mg/kg per day produced the strongest effect, which was comparable to that of 4.75 mg/kg/per day or 19 mg/kg/per day.

### 3.4. Immunohistochemistry Results

**Figure 3:**
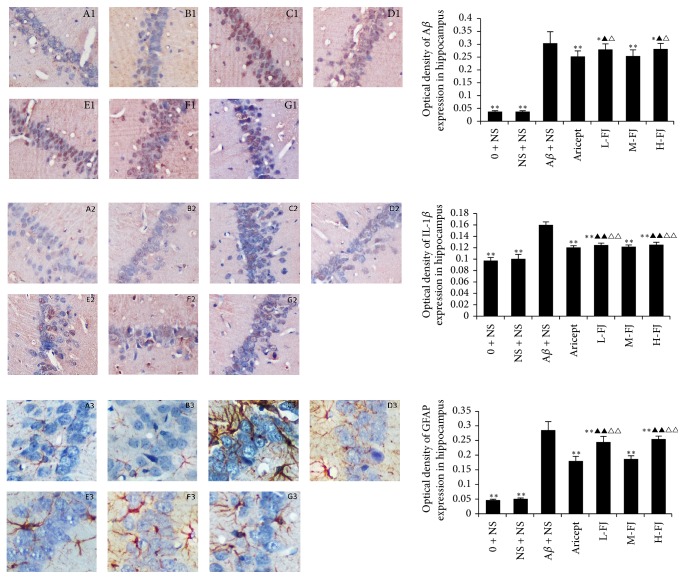
Expression of A*β*, IL-1*β*, and GFAP in the hippocampus (×200 magnification). Compared to A*β* + NS group, ^*∗∗*^*P* < 0.01, ^*∗*^*P* < 0.05; compared to Aricept group, ^△△^*P* < 0.01, ^△^*P* < 0.05; compared to “M-FJ” group, ^▲^*P* < 0.05, ^▲▲^*P* < 0.01. A: normal group, B: sham operation group, C: model group, D: Aricept group, E: low-dose group, F: moderate-dose group, and G: high-dose group.

The expression levels of A*β*, IL-1*β*, and GFAP in the hippocampus were significantly increased in the AD model group (*P* < 0.01; Figure 3) in comparison to the other groups. After treatment with donepezil or QKF, changes in A*β*, IL-1*β*, and GFAP protein levels were reversed. Of the QKF treatments, a dose of 9.5 mg/kg per day produced the strongest effect, which was comparable to that of 4.75 mg/kg/per day or 19 mg/kg/per day.

### 3.5. Methenamine Silver Granule Staining Results

**Figure 4:**
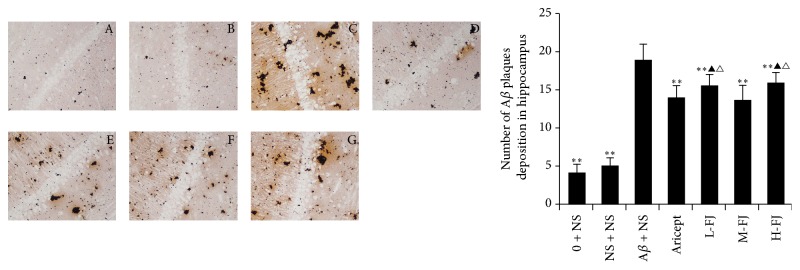
Methenamine sliver granules staining results. Compared to A*β* + NS group, ^*∗∗*^*P* < 0.01; compared to Aricept group, ^△^*P* < 0.05; compared to “M-FJ” group, ^▲^*P* < 0.05. A: normal group, B: sham operation group, C: model group, D: Aricept group, E: low-dose group, F: moderate-dose group, and G: high-dose group.

Methenamine silver granule staining showed no significant differences between the control group and the sham-operated group (Figure 4). Methenamine silver staining was significantly increased in the model group in comparison to the other groups (*P* < 0.05, *P* < 0.01). After treatment with donepezil or QKF, changes in methenamine silver staining were reduced compared to the model group (*P* < 0.05, *P* < 0.01); however, there was no significant difference between the moderate-dose group and the donepezil group (*P* > 0.05) (Figure 4).

## 4. Discussion

Alzheimer's disease (AD) is the most common form of dementia and causes problems with memory, cognition, and behavior. The incidence of AD increases with age, and AD seriously affects the health and quality of life of affected individuals, their families, and their communities. Currently, the main methods for AD treatment in China and worldwide are based in Western medicine methods and rehabilitation. The former causes undesirable side effects and the latter is expensive and has variable outcomes. Treatments based in traditional Chinese medicine (TCM) have shown potential to treat AD, and many studies have reported on the use of TCM for AD treatment in clinical practice [12, 13].

The etiology and molecular mechanisms of AD are complex and not well understood [14]. Hypotheses for AD pathogenesis include A*β* cascade [15, 16], immune responses with inflammation [17, 18], cholinergic defects [19, 20], tau protein hyperphosphorylation [21, 22], intracellular calcium homeostasis disorders [23, 24], and peroxidation [25]. These hypotheses are indicative of the complex nature of AD. However, A*β* deposition in neurons in the brain is well known to be an initial event in AD [26] and a key trigger of AD pathogenesis. A*β* causes neurons in the brain to undergo apoptosis [27], which results in a series of consequences such as glial cell activation [28], triggering of the inflammatory cascade [29], oxidative stress [30, 31], and excessive expression of NO [32].

A large number of studies have shown that inflammation is associated with AD. A*β* deposition is caused by microglia, astrocytes, inflammatory cytokines, free radicals, and chemokines that play a major role in the inflammatory response. Activated glial cells accumulation is caused by inflammatory cytokines [33], which may play an important role in the pathology of AD. Proinflammatory cytokines, such as IL-1*β*, and IL-6, are elevated in the brain and cerebrospinal fluid of patients with AD [34]. IL-1 family cytokines have been shown to induce endothelial cell amyloid precursor protein (APP) mRNA expression [35], and thus increased levels of IL-1 cytokines in the brains of AD patients might be related to A*β* formation.

In the early stages of AD, astrocytes in the molecular layer of the cerebral cortex are activated by A*β* sediments. Astrocyte activation is associated with high expression of glial fibrillary acidic protein (GFAP) and local neuronal depolarization, which can contribute to brain injury. It has been shown that injecting oligomeric A*β* into the rat cortex can cause significant changes in astrocytes, including activation of transcription factor NF-*κ*B and inflammatory mediators such as TNF-*α* and IL-1*β* [36]. IL-1 cytokines have been shown to upregulate APP in neurons by stimulating its promoter, which promotes the formation of SP [37]. Therefore, the activation of astrocytes has protective effects on the brain, but excessive activation leads to nerve injury and accelerates the development of neurodegenerative diseases.

## 5. Conclusions

In the present study, we established AD model in rats by bilateral injecting A*β* 1–40 into the hippocampus and used this AD model to test the effects of TCM compound QKF. We were able to reliably establish AD model in rats by A*β* 1–40 injection. In AD model rats, after treating with QKF at various doses for 14 days, QKF-treated rats showed decreased IL-1*β*, GFAP, and A*β* expression in the hippocampus. The efficacy of QKF at 9.5 mg/kg/day was similar to that of the positive control donepezil, and both treatments produced significant differences compared to the model group (*P* < 0.01 and *P* < 0.05, respectively). QKF may be effective in the treatment of AD by having effect on IL-1*β*, GFAP, and A*β*.

## Figures and Tables

**Table 1:** Polymerase chain reaction primers.

Primers	Sequence (5′-3′)
A*β*	Forward CTGGAGGTGCCCACTGATG
	Reverse GGGTCTGACTCCCATTTTCC
IL-1*β*	Forward TCTGTGACTCGTGGGATGATGAC
	Reverse TTGGCTTATGTTCTGTCCATTGAG
GFAP	Forward AGAGTGGTATCGGTCCAAGTT
	Reverse TCAAGGTCGCAGGTCAAG
*β*-Actin	Forward CCCATCTATGAGGGTTACGC
	Reverse TTTAATGTCACGCACGATTTC

## References

[1] Hebert L. E., Scherr P. A., Bienias J. L., Bennett D. A., Evans D. A. (2003). Alzheimer disease in the US population: prevalence estimates using the 2000 census. *JAMA Neurology*.

[2] Maccioni R. B., Muñoz J. P., Barbeito L. (2001). The molecular bases of Alzheimer's disease and other neurodegenerative disorders. *Archives of Medical Research*.

[3] Nicoll J. A. R., Yamada M., Frackowiak J., Mazur-Kolecka B., Weller R. O. (2004). Cerebral amyloid angiopathy plays a direct role in the pathogenesis of Alzheimer's disease: Pro-CAA position statement. *Neurobiology of Aging*.

[4] Gorman A. M. (2008). Neuronal cell death in neurodegenerative diseases: recurring themes around protein handling: Apoptosis Review Series. *Journal of Cellular and Molecular Medicine*.

[5] Butterfield D. A., Griffin S., Munch G., Pasinetti G. M. (2002). Amyloid *β*-peptide and amyloid pathology are central to the oxidative stress and inflammatory cascades under which Alzheimer's disease brain exists. *Journal of Alzheimer's Disease*.

[6] Bamberger M. E., Harris M. E., McDonald D. R., Husemann J., Landreth G. E. (2003). A cell surface receptor complex for fibrillar *β*-amyloid mediates microglial activation. *The Journal of Neuroscience*.

[7] Hong W., Zhang J. Y. (2000). Academic thoughts about dementia. *Hebei J TCM (China)*.

[8] Yan Q. L., Xing B. (2003). Development of TCM treatment in Alzheimer's disease objective and method. *JTCM (China)*.

[9] Wang Y. F., Yan Q. L. (2004). Uses the clearing method in the treatment of Alzheimers disease experience. *Jiangsu J Tradit Chin Med (China)*.

[10] Zhang Y. L., Liou D. M., QS W. u., Yao Y. Y., Li W. P. (2007). *Effect of the extract of astragalus on learning and memory and the expression of Bcl-2 and Bcl-xl protein in hippocampus neurons in rat model with Alzheimer's Disease*.

[11] George P., Charles W. (2007). *The rat brain in stereotaxic coordinates*.

[12] Hu H. Y., Meng Q., Jiang Z., Zhu W. M., Wang W. H., Zhang X. Y. (2008). Effect of Qingxin Kaiqiao Fang on learning and memory ability and morphology of hippocampal nerve cells in AD mice. *China Journal of Chinese Material Medico (China)*.

[13] Zhu W. M., Meng Q., Jiang Z., HY H. u., Wang W. H., Zhang X. Y. (2008). Effect of Qingxin Kaiqiaofang treatment in AD mice on learning and memory ability as well as the contents of NO AchE in mouse brain. *Chinese Archives of Traditional Chinese Medicine (China)*.

[14] Hong T. (2001). *Infectious & Non-Infectious Dementia Prion & Alzheimers Disease*.

[15] Zhao W.-Q., de Felice F. G., Fernandez S. (2008). Amyloid beta oligomers induce impairment of neuronal insulin receptors. *The FASEB Journal*.

[16] Viola K. L., Velasco P. T., Klein W. L. (2008). Why Alzheimer’s disease is a disease of memory: the attack on synapses by A-beta oligomers (ADDLs). *The Journal of Nutrition, Health & Aging*.

[17] Vasto S., Candore G., Listì F. (2008). Inflammation, genes and zinc in Alzheimer's disease. *Brain Research Reviews*.

[18] Ojala J., Alafuzoff I., Herukka S.-K., van Groen T., Tanila H., Pirttilä T. (2009). Expression of interleukin-18 is increased in the brains of Alzheimer's disease patients. *Neurobiology of Aging*.

[19] Gulledge A. T., Stuart G. J. (2005). Cholinergic inhibition of neocortical pyramidal neurons. *The Journal of Neuroscience*.

[20] Watanabe T., Yamagata N., Takasaki K. (2009). Decreased acetylcholine release is correlated to memory impairment in the Tg2576 transgenic mouse model of Alzheimer's disease. *Brain Research*.

[21] Muntané G., Dalfó E., Martinez A., Ferrer I. (2008). Phosphorylation of tau and *α*-synuclein in synaptic-enriched fractions of the frontal cortex in Alzheimer's disease, and in Parkinson's disease and related *α*-synucleinopathies. *Neuroscience*.

[22] Hanger D. P., Anderton B. H., Noble W. (2009). Tau phosphorylation: the therapeutic challenge for neurodegenerative disease. *Trends in Molecular Medicine*.

[23] Thibault O., Gant J. C., Landfield P. W. (2007). Expansion of the calcium hypothesis of brain aging and Alzheimer's disease: minding the store. *Aging Cell*.

[24] Cheung K.-H., Shineman D., Müller M. (2008). Mechanism of Ca2+ Disruption in Alzheimer's Disease by Presenilin Regulation of InsP3 Receptor Channel Gating. *Neuron*.

[25] Reed T., Perluigi M., Sultana R. (2008). Redox proteomic identification of 4-Hydroxy-2-nonenal-modified brain proteins in amnestic mild cognitive impairment: Insight into the role of lipid peroxidation in the progression and pathogenesis of Alzheimer's disease. *Neurobiology of Disease*.

[26] Moir R. D., Tanzi R. E. (2005). LRP-mediated clearance of A*β* is inhibited by KPI-containing isoforms of APP. *Current Alzheimer Research*.

[27] Zhang H. S., Lin Q., Yang D. H. (2009). Research progress of a ß toxicity mechanism of Alzheimer's disease. *China Modern Medicine*.

[28] Stein T. D., Anders N. J., DeCarli C., Chan S. L., Mattson M. P., Johnson J. A. (2004). Neutralization of transthyretin reverses the neuroprotective effects of secreted amyloid precursor protein (APP) in APPSw mice resulting in tau phosphorylation and loss of hippocampal neurons: Support for the amyloid hypothesis. *The Journal of Neuroscience*.

[29] Streit W. J. (2004). Microglia and Alzheimer's disease pathogenesis. *Journal of Neuroscience Research*.

[30] Klyubin I., Walsh D. M., Lemere C. A. (2005). Amyloid *β* protein immunotherapy neutralizes A*β* oligomers that disrupt synaptic plasticity in vivo. *Nature Medicine*.

[31] Ma G. S., Chen S. D., Ba M. W. (2005). A*β* Induced Glioma cell U251 protein aggregates formation of oxygen content and mitochondrial membrane potential increase. *Chin J Neural*.

[32] Rowan M. J., Klyubin I., Wang Q., Anwyl R. (2005). Synaptic plasticity disruption by amyloid *β* protein: Modulation by potential Alzheimer's disease modifying therapies. *Biochemical Society Transactions*.

[33] Nagele R. G., D'Andrea M. R., Lee H., Venkataraman V., Wang H.-Y. (2003). Astrocytes accumulate A*β*42 and give rise to astrocytic amyloid plaques in Alzheimer disease brains. *Brain Research*.

[34] Blum-Degen D., Müller T., Kuhn W. (1995). Interleukin-1 beta and interleukin-6 are elevated in the cerebrospinal fluid of Alzheimer’s and de novo Parkinson’s disease patients. *Neuroscience Letters*.

[35] Goldgaber D., Harris H. W., Hla T. (1989). Interleukin 1 regulates synthesis of amyloid *β*-protein precursor mRNA in human endothelial cells. *Proceedings of the National Acadamy of Sciences of the United States of America*.

[36] Halassa M. M., Haydon P. G. (2010). Integrated brain circuits: astrocytic networks modulate neuronal activity and behavior. *Annual Review of Physiology*.

[37] Chiang C. S., Stalder A., Samimi A., Campbell I. L. (1994). Reactive gliosis as a consequence of interleukin-6 expression in the brain: studies in transgenic mice. *Developmental Neuroscience*.

